# Heterogeneity of tissue-resident immunity across organs and in health and disease

**DOI:** 10.1007/s00281-022-00967-z

**Published:** 2022-10-28

**Authors:** Petra Clara Arck, Federica Sallusto

**Affiliations:** 1 Division of Experimental Feto-Maternal Medicine, Department of Obstetrics and Fetal Medicine, University Medical Center Hamburg-Eppendorf, 20246 Hamburg, Germany; 2 Institute for Research in Biomedicine, Università Della Svizzera Italiana, 6500 Bellinzona, Switzerland; 3 Institute of Microbiology, ETH Zürich, 8093 Zurich, Switzerland

The conception of how the immune system is operational has been significantly better understood in recent years. One critical finding is the recognition that lymphocytes are not necessarily constantly recirculating throughout the body, as subsets of T lymphocytes reside at certain tissue sites. Consequently, the notion of tissue-resident immunity has evolved. In the present special issue, we compiled chapters that comprehensively define the role of resident immune cells at various sites and settings. Feyaerts et al. discuss current knowledge about the development and function of tissue-resident immune populations during fetal life, focusing on the brain, lung, and gastrointestinal tract as sites with distinct developmental trajectories [1]. Haertel and colleagues build on this knowledge related to the developmental origin of tissue-resident immunity and review its role in children, including in highly vulnerable preterm born children. For example, tissue-resident memory T cells are diminished in airway tissues of neonates, compared to older children or adults, which hampers the ability to make specific recall responses after secondary infectious challenges. They introduce the microbiome as a dominant factor in shaping resident immunity at mucosal surfaces. The microbiome is often disturbed in preterm born infants, which can increase the risk for tissue inflammation in these children. They emphasize that an improved understanding of tissue-resident immunity holds the potential to unearth novel targets of vaccination and enables individualized approaches to protect preterm born babies in the future [2] .

In adults, the knowledge of tissue-resident immunity in different organs greatly varies. For examples, in the female or male reproductive tracts, insights on tissue-resident immunity are sparse. This is surprising, since the need for protection from vaginal and amniotic infections or the necessity to immunologically adapt to the semiallogenic fetus during pregnancy underscores the relevance of tissue-resident immunity in the female reproductive tract. Yüzen et al. review the current knowledge of uterine tissue-resident immunity in modulating the risk for infertility, pregnancy complications, infections, or cancer and outline the still open questions. They also summarize the evidence published to date on tissue-resident immunity in the male reproductive organs, which is still a largely uncharted territory [3] .

In other organs in adults, tissue-resident immunity seems to be better understood, for example, in the kidneys. Thus, Asada et al. propose therapeutic options resulting from the insights on resident immune cells in the kidneys available to date, which include emerging treatment options for kidney infections, autoimmune diseases, graft rejection, and cancer [4]. In the liver, memory T cells with a profile of tissue residency have been identified. Pallett and Maini review how these cells are retained in the liver and describe their potential interactions with other local cell types. These cells may be functionally critical in hepatotropic infections affecting individuals worldwide, such as hepatitis B or malaria, as well as in hepatocellular carcinoma. Thus, the authors propose that monitoring these cells may enable to accurate assess disease activity. Also, the understanding of memory T cells in the liver opens avenues for locally targeted immunotherapies [5]. In the lung—an organ that incessantly faces external environmental challenges—homeostasis and function are ensured by the respiratory epithelium. Zazara et al. discuss how tissue-resident immune cells form an intricate network with the respiratory epithelium. Functionally, tissue-resident immune cells in the lung are known to protect from infectious agents. Conversely, if dysregulated, lung-resident immunity can contribute to the pathogenesis of respiratory diseases [6].

In the brain, understanding the role for resident T cells has been challenged by the compartmentalized organization of the central nervous system (CNS). Hamann and colleagues review the infiltration, phenotypic characteristics, and functions of T cells in the cerebrospinal fluid, the perivascular space, the meninges, and the parenchyma. Many of these insights have arisen from studies in the context of autoimmunity, i.e., multiple sclerosis (MS). The authors propose that a better understanding of the dynamics of physiological CNS surveillance by T cells can improve the understanding of pathological conditions, such as MS [7]. Carloni and Rescigno discuss the influence of the tissue-specific vascular unit in the gut in establishing immune homeostasis and response to systemic stimuli. They suggest that the choroid plexus gatekeeper becomes a second barrier which protects the CNS from systemic inflammation in case the gut vascular barrier is compromised [8]. Notarbartolo and Abrignani review the generation and role of resident memory T cells in antitumor immunity. These T cells persist as long-lived memory cells and generate enhanced immune responses when re-encountering antigens. Thus, tissue-resident memory T cells are potentially endowed with the capacity to protect against the reemergence of cancer cells. They also hold the possibility to contribute to the efficacy of immunotherapies [9].

Taken together, the propensity to adapt to the local microenvironments in various organs and settings renders tissue-resident immune cells as professional tissue defenders, e.g., upon pathogen challenge in the uterus, gut, liver, lung, and other organs. Emerging insights also underpin the importance of tissue-resident immune cells in antitumor immunity, especially in the first stages of tumor development. Their potential to enhance the efficacy of immunotherapies and vaccinations in various settings highlights the need to systematically dissect the function of the different immune cell subsets at various sites. High-throughput technologies, e.g., multiparametric flow, mass cytometry, spatial transcriptomics, and single-cell RNA and T cell receptor sequencing, will aid to acquire the urgently needed insights to promote the development of novel targeted immunotherapies.
